# Supplementary material for: Improving Maximum Tolerated Dose Selection in Model‐Assisted Designs for Phase I Trials Through Bayesian Dose–Response Model
Source: Pharm Stat. 2025 Oct 25;24(6):e70046. doi: 10.1002/pst.70046 (PMC12553356; doi:10.1002/pst.70046)
Supplement: Supplementary file 1 — Data S1: pst70046‐sup‐0001‐Supinfo.pdf. [file PST-24-0-s001.pdf]

Supplementary Material of "Improving Maximum Tolerated Dose Selection in Model-Assisted Designs for Phase I Trials through Bayesian Dose-Response Model"

Rentaro Wakayama[1], Tomotaka Momozaki[2], and Shuji Ando[2]

[1]Department of Information Sciences, Graduate School of Science and Technology, Tokyo University of Science
[2]Department of Information Sciences, Faculty of Science and Technology, Tokyo University of Science

Last update: August 15, 2025

A Sensitivity Analyses for Prior Specification

To assess the robustness of our proposed method to prior specification choices, we conducted sensitivity analyses using the same simulation settings as described in Section 4.1 of the main manuscript. All simulations were conducted under identical conditions to the main study: 3 patients per cohort, 12 cohorts (36 participants total), six dose levels (10, 20, 30, 45, 60, 80) mg, target DLT probability $\phi = 0.3$, and the same eight DLT probability scenarios (for details, see Table 3 in the main manuscript). We applied the Bayesian dose-response model,

$$g(\pi(d_j)) = \beta_0 + \exp(\beta_1) \log\left(\frac{d_j}{d^*}\right), \tag{1}$$

where $g(\cdot)$ is the link function, β_0 and β_1 are coefficient parameters, and $\pi(d_j)$ is the DLT probability for dose d_j. The d^*, referred to as the reference dose, is selected from $\{d_j\}$ (e.g., $d^* = d_3$). We examined two types of prior specification using logit, log-log, and complementary log-log link functions: (1) alternative specifications of p_1 and p_J values that differ from our BOIN-aligned defaults, and (2) large variance normal priors for coefficient parameters that lead to inappropriate U-shaped prior distributions for intermediate doses. Note that Bayesian inference was performed using the same computational approach as described in Section 4.2, employing the `cmdstanr` package with 2500 samples (500 burn-in, 2000 for posterior estimation).

A.1 Alternative specifications of p_1 and p_J values

We examined the impact of alternative specifications for the probability values $p_1 = P\{\pi(d_1) > 0.3\}$ and $p_J = P\{\pi(d_J) \leq 0.3\}$ that differ from our BOIN-aligned defaults used in the main analysis. As described in Section 3.2 of the main manuscript, our default specification sets $p_1 = 0.05$ for the lowest dose and $p_J = 0.21$ (corresponding to $P\{\pi(d_J) \leq 0.1\} = 0.05$) for the highest dose, which aligns with BOIN's safety mechanisms and addresses the dual concerns of controlling unacceptable toxicity risk while avoiding underdosing. For p_1, we considered four alternative values: $p_1 = 0.09$ (corresponding to $P\{\pi(d_1) > \phi + \Delta_U\} = 0.05$, where $\phi + \Delta_U = 0.3585$ represents

the BOIN escalation boundary); $p_1 = 0.2$ (representing a moderately conservative approach); $p_1 = 0.31$ (corresponding to $P\{\pi(d_1) > 0.6\} = 0.05$, which is the default setting of Neuenschwander et al., 2008); and $p_1 = 0.7$ (corresponding to $\pi(d_1) \sim \text{Beta}(1, 1)$, representing a uniform prior). For p_J, we examined three alternative values: $p_J = 0.08$ (corresponding to $P\{\pi(d_J) \leq \phi - \Delta_L\} = 0.05$, where $\phi - \Delta_L = 0.2365$ represents the BOIN de-escalation boundary), $p_J = 0.11$ (corresponding to $P\{\pi(d_J) \leq 0.2\} = 0.05$, the default setting from Neuenschwander et al., 2008), and $p_J = 0.3$ (corresponding to $\pi(d_J) \sim \text{Beta}(1, 1)$). To isolate the individual effects of each parameter, we conducted separate sensitivity analyses, that is, when examining p_1 sensitivity, we fixed $p_J = 0.21$ (our default); when examining p_J sensitivity, we fixed $p_1 = 0.05$ (our default). For each alternative specification, we followed the same procedure described in Section 3.2 to derive the corresponding prior distributions for the coefficient parameters β_0 and β_1 through the optimization process that minimizes the discrepancy between target quantiles and model-implied quantiles.

Tables A.1-A.7 present the MTD selection results under alternative specifications of p_1 and p_J values across all eight scenarios. Comparing these results with Table 4 in the main manuscript (our default specification: $p_1 = 0.05$, $p_J = 0.21$) reveals several important sensitivity patterns.

For p_1 sensitivity analysis (Tables A.1-A.4, with p_J fixed at 0.21), the results demonstrate that our method is generally robust to moderate variations in p_1 specification. The alternative specifications $p_1 = 0.09$, $p_1 = 0.2$, and $p_1 = 0.31$ show minimal differences compared to our default specification ($p_1 = 0.05$), with the MTD selection accuracy remaining largely stable in most scenarios. However, the extreme specification $p_1 = 0.7$ (corresponding to a uniform prior) shows notable performance deterioration, particularly in scenarios with intermediate MTDs. In Scenarios 1, 3, 4, 5, 6, 7, and 8, the logit model shows an average decrease of approximately 8 percentage points in the correct MTD selection rate. More concerning, some link functions experience reductions exceeding about 20 percentage points. For example, in Scenario 8, the log-log model decreases from 57.4% to 36.8% (a 20.6 percentage point reduction), and the clog-log model decreases from 60.3% to 40.8% (a 19.5 percentage point reduction). In contrast, Scenario 2 (where the MTD is at the highest dose level) shows improved performance with $p_1 = 0.7$. The logit model increases from 51.6% to 61.1% (a 9.5 percentage point improvement), with even larger improvements observed for the log-log model (43.7% to 64.0%, a 20.3 percentage point increase). This improvement likely occurs because the specification $p_1 = 0.7$ implies that the lowest dose is expected to have relatively high toxicity. Through the dose-response model, this translates to a prior distribution that accommodates higher DLT probabilities at higher doses. This may lead to more aggressive dose selection that favors higher doses, which aligns well with Scenario 2 where the true MTD is at the highest dose level.

For p_J sensitivity analysis (Tables A.5-A.7, with p_1 fixed at 0.05), the results demonstrate that our method shows greater robustness to variations in p_J specification compared to p_1 variations. All alternative specifications show minimal impact on MTD selection accuracy across most scenarios, with differences typically within approximately 1-3 percentage points compared to our default specification ($p_J = 0.21$). However, one notable exception occurs with $p_J = 0.08$ in Scenario 2 (MTD at the highest dose level), where the logit model shows a substantial decrease from 51.6% to 43.6% (an 8.0 percentage point reduction). This deterioration likely occurs because the more conservative specification $p_J = 0.08$ implies lower expected toxicity at the highest dose, which may discourage selection of higher doses even when they represent the true MTD.

The relatively lower sensitivity to p_J variations compared to p_1 variations suggests that the specification of prior beliefs about toxicity at the lowest dose level has a more

substantial impact on overall model behavior than beliefs about the highest dose level. This finding aligns with the clinical importance of establishing appropriate safety constraints at the lowest dose level in phase I trials. Across both p_1 and p_J sensitivity analyses, the patterns remain consistent across all three link functions (logit, log-log, and complementary log-log), demonstrating that the sensitivity patterns are inherent to the prior specification approach rather than dependent on the choice of link function.

Table A.1: MTD selection results for $p_1 = 0.09$ with $p_J = 0.21$. Prior distributions: logit: $\beta_0 \sim N(-1.067, 1.179)$, $\beta_1 \sim N(0.219, 0.898)$; log-log: $\beta_0 \sim N(-0.196, 1.217)$, $\beta_1 \sim N(0.328, 0.569)$; clog-log: $\beta_0 \sim N(-1.255, 0.932)$, $\beta_1 \sim N(0.092, 0.642)$.

Dose levels		1	2	3	4	5	6
Doses		10	20	30	45	60	80
scenario 1	PAVA	0.9	9.2	28.1	**33.8**	24.6	3.4
	logit	0.0	7.4	29.4	**39.0**	19.9	4.3
	log-log	0.0	9.5	32.9	**40.4**	16.2	1.0
	clog-log	0.0	7.4	28.5	**39.8**	20.7	3.6
scenario 2	PAVA	0.0	0.7	7.4	14.6	26.6	**50.7**
	logit	0.0	0.0	7.5	14.3	26.0	**52.2**
	log-log	0.0	0.0	7.6	19.6	31.0	**41.8**
	clog-log	0.0	0.0	6.9	15.1	26.9	**51.1**
scenario 3	PAVA	0.0	0.2	6.9	27.4	**56.7**	8.8
	logit	0.0	0.0	5.4	31.4	**51.5**	11.7
	log-log	0.0	0.0	5.7	36.5	**52.9**	4.9
	clog-log	0.0	0.0	4.5	32.2	**52.8**	10.5
scenario 4	PAVA	19.6	**46.8**	27.2	4.5	0.8	0.0
	logit	8.2	**56.4**	29.2	5.0	0.1	0.0
	log-log	15.7	**56.8**	23.8	2.5	0.1	0.0
	clog-log	7.3	**56.4**	30.0	5.1	0.1	0.0
scenario 5	PAVA	3.1	29.2	**51.0**	14.1	2.4	0.1
	logit	0.2	24.3	**59.4**	14.7	1.2	0.1
	log-log	1.2	32.9	**53.3**	11.7	0.8	0.0
	clog-log	0.2	23.9	**58.8**	15.7	1.2	0.1
scenario 6	PAVA	0.2	2.6	26.3	**49.4**	19.6	1.9
	logit	0.0	0.9	26.1	**53.8**	17.0	2.2
	log-log	0.0	1.4	30.9	**54.6**	12.5	0.6
	clog-log	0.0	0.9	24.5	**55.5**	17.4	1.7
scenario 7	PAVA	20.0	**61.9**	16.9	1.1	0.0	0.0
	logit	2.4	**74.0**	22.9	0.6	0.0	0.0
	log-log	6.7	**76.4**	16.5	0.3	0.0	0.0
	clog-log	1.8	**73.6**	23.8	0.7	0.0	0.0
scenario 8	PAVA	3.1	29.5	**52.7**	13.3	1.3	0.0
	logit	0.2	24.5	**61.5**	13.2	0.5	0.0
	log-log	1.2	33.4	**55.3**	9.8	0.2	0.0
	clog-log	0.2	24.0	**61.0**	14.2	0.5	0.0

Table A.2: MTD selection results for $p_1 = 0.20$ with $p_J = 0.21$. Prior distributions: logit: $\beta_0 \sim N(-0.945, 1.402)$, $\beta_1 \sim N(0.103, 0.742)$; log-log: $\beta_0 \sim N(0.008, 0.867)$, $\beta_1 \sim N(-0.160, 0.500)$; clog-log: $\beta_0 \sim N(-1.121, 0.937)$, $\beta_1 \sim N(-0.165, 0.661)$.

Dose levels		1	2	3	4	5	6
Doses		10	20	30	45	60	80
scenario 1	PAVA	0.9	9.2	28.1	**33.8**	24.6	3.4
	logit	0.0	7.6	28.2	**39.4**	20.3	4.5
	log-log	0.0	8.5	30.4	**42.2**	17.0	1.9
	clog-log	0.0	7.4	26.6	**40.4**	21.0	4.6
scenario 2	PAVA	0.0	0.7	7.4	14.6	26.6	**50.7**
	logit	0.0	0.0	6.9	14.3	26.0	**52.8**
	log-log	0.0	0.0	6.5	18.6	29.6	**45.3**
	clog-log	0.0	0.0	6.4	15.0	25.5	**53.1**
scenario 3	PAVA	0.0	0.2	6.9	27.4	**56.7**	8.8
	logit	0.0	0.0	4.4	30.0	**53.5**	12.1
	log-log	0.0	0.0	4.1	36.7	**51.6**	7.6
	clog-log	0.0	0.0	4.2	30.4	**53.3**	12.1
scenario 4	PAVA	19.6	**46.8**	27.2	4.5	0.8	0.0
	logit	9.9	**56.0**	28.1	4.8	0.1	0.0
	log-log	12.8	**58.7**	24.5	2.8	0.1	0.0
	clog-log	8.3	**56.2**	29.1	5.2	0.1	0.0
scenario 5	PAVA	3.1	29.2	**51.0**	14.1	2.4	0.1
	logit	0.2	25.4	**58.0**	14.9	1.3	0.1
	log-log	0.5	31.5	**55.1**	12.0	0.8	0.0
	clog-log	0.2	24.2	**57.7**	16.4	1.3	0.1
scenario 6	PAVA	0.2	2.6	26.3	**49.4**	19.6	1.9
	logit	0.0	0.9	24.6	**54.1**	18.2	2.2
	log-log	0.0	0.9	27.8	**56.8**	13.7	0.8
	clog-log	0.0	0.9	21.7	**57.0**	18.2	2.2
scenario 7	PAVA	20.0	**61.9**	16.9	1.1	0.0	0.0
	logit	2.9	**75.2**	21.1	0.7	0.0	0.0
	log-log	4.3	**77.8**	17.5	0.3	0.0	0.0
	clog-log	2.4	**74.5**	22.1	0.9	0.0	0.0
scenario 8	PAVA	3.1	29.5	**52.7**	13.3	1.3	0.0
	logit	0.2	25.8	**59.9**	13.4	0.6	0.0
	log-log	0.5	32.0	**57.0**	10.2	0.2	0.0
	clog-log	0.2	24.4	**59.9**	14.7	0.7	0.0

Table A.3: MTD selection results for $p_1 = 0.31$ with $p_J = 0.21$. Prior distributions: logit: $\beta_0 \sim N(-0.599, 1.515)$, $\beta_1 \sim N(-0.124, 0.500)$; log-log: $\beta_0 \sim N(0.143, 0.824)$, $\beta_1 \sim N(-1.176, 1.470)$; clog-log: $\beta_0 \sim N(-0.991, 1.020)$, $\beta_1 \sim N(-0.447, 0.542)$.

Dose levels		1	2	3	4	5	6
Doses		10	20	30	45	60	80
scenario 1	PAVA	0.9	9.2	28.1	**33.8**	24.6	3.4
	logit	0.0	6.8	26.1	**39.3**	22.3	5.5
	log-log	0.0	8.8	29.0	**37.4**	19.6	5.2
	clog-log	0.0	6.8	24.3	**40.2**	22.8	5.9
scenario 2	PAVA	0.0	0.7	7.4	14.6	26.6	**50.7**
	logit	0.0	0.0	5.3	15.5	23.4	**55.8**
	log-log	0.0	0.2	7.6	13.8	23.8	**54.6**
	clog-log	0.0	0.0	5.1	15.6	22.9	**56.4**
scenario 3	PAVA	0.0	0.2	6.9	27.4	**56.7**	8.8
	logit	0.0	0.0	3.0	28.7	**51.4**	16.9
	log-log	0.0	0.0	6.5	29.8	**49.6**	14.1
	clog-log	0.0	0.0	2.8	28.7	**50.7**	17.8
scenario 4	PAVA	19.6	**46.8**	27.2	4.5	0.8	0.0
	logit	11.3	**56.7**	25.9	4.9	0.1	0.0
	log-log	16.0	**55.0**	23.8	3.7	0.4	0.0
	clog-log	10.6	**55.9**	27.2	5.0	0.2	0.0
scenario 5	PAVA	3.1	29.2	**51.0**	14.1	2.4	0.1
	logit	0.3	26.0	**55.3**	16.5	1.7	0.1
	log-log	1.2	31.3	**53.5**	12.1	1.7	0.1
	clog-log	0.3	24.5	**55.9**	17.2	1.9	0.1
scenario 6	PAVA	0.2	2.6	26.3	**49.4**	19.6	1.9
	logit	0.0	1.1	20.9	**54.7**	20.7	2.6
	log-log	0.0	1.8	27.8	**49.8**	18.0	2.6
	clog-log	0.0	1.0	20.0	**54.1**	21.7	3.2
scenario 7	PAVA	20.0	**61.9**	16.9	1.1	0.0	0.0
	logit	4.0	**75.4**	19.9	0.6	0.0	0.0
	log-log	6.6	**76.6**	16.1	0.6	0.0	0.0
	clog-log	3.8	**74.7**	20.4	1.0	0.0	0.0
scenario 8	PAVA	3.1	29.5	**52.7**	13.3	1.3	0.0
	logit	0.3	26.6	**57.2**	15.0	0.8	0.0
	log-log	1.1	32.0	**55.8**	10.2	0.8	0.0
	clog-log	0.3	25.1	**57.9**	15.8	0.8	0.0

Table A.4: MTD selection results for $p_1 = 0.7$ with $p_J = 0.21$. Prior distributions: logit: $\beta_0 \sim N(0.074, 1.713)$, $\beta_1 \sim N(-1.638, 1.087)$; log-log: $\beta_0 \sim N(0.410, 1.412)$, $\beta_1 \sim N(-2.330, 0.543)$; clog-log: $\beta_0 \sim N(-0.340, 1.532)$, $\beta_1 \sim N(-1.512, 0.500)$.

Dose levels		1	2	3	4	5	6
Doses		10	20	30	45	60	80
scenario 1	PAVA	0.9	9.2	28.1	**33.8**	24.6	3.4
	logit	0.1	7.4	23.0	**36.3**	23.1	10.1
	log-log	1.6	8.1	17.6	**33.9**	23.6	15.2
	clog-log	0.2	7.8	19.3	**35.8**	22.4	14.5
scenario 2	PAVA	0.0	0.7	7.4	14.6	26.6	**50.7**
	logit	0.0	0.0	4.9	13.9	20.1	**61.1**
	log-log	0.0	0.0	4.5	13.0	18.5	**64.0**
	clog-log	0.0	0.0	4.3	13.4	18.4	**63.9**
scenario 3	PAVA	0.0	0.2	6.9	27.4	**56.7**	8.8
	logit	0.0	0.0	2.9	25.5	**42.4**	29.2
	log-log	0.1	0.9	3.5	17.8	**37.8**	39.9
	clog-log	0.0	0.3	3.1	20.7	**36.6**	39.3
scenario 4	PAVA	19.6	**46.8**	27.2	4.5	0.8	0.0
	logit	20.0	**49.2**	22.7	6.2	0.8	0.0
	log-log	32.2	**36.6**	20.6	8.1	1.3	0.1
	clog-log	24.0	**44.2**	21.8	7.8	1.0	0.1
scenario 5	PAVA	3.1	29.2	**51.0**	14.1	2.4	0.1
	logit	1.8	27.0	**49.1**	18.4	3.5	0.1
	log-log	10.8	21.5	**36.8**	25.5	4.5	0.8
	clog-log	5.1	24.7	**40.9**	24.3	4.3	0.6
scenario 6	PAVA	0.2	2.6	26.3	**49.4**	19.6	1.9
	logit	0.0	1.6	18.7	**48.3**	25.0	6.4
	log-log	1.4	4.0	13.3	**41.3**	29.8	10.2
	clog-log	0.2	2.7	16.0	**43.6**	27.7	9.8
scenario 7	PAVA	20.0	**61.9**	16.9	1.1	0.0	0.0
	logit	14.5	**66.1**	17.9	1.3	0.1	0.0
	log-log	33.0	**44.1**	20.7	1.9	0.2	0.0
	clog-log	23.3	**53.5**	21.3	1.7	0.1	0.0
scenario 8	PAVA	3.1	29.5	**52.7**	13.3	1.3	0.0
	logit	1.8	27.8	**50.4**	18.1	1.8	0.0
	log-log	11.5	22.4	**36.8**	25.6	3.5	0.1
	clog-log	5.3	26.2	**40.8**	24.4	3.1	0.1

Table A.5: MTD selection results for $p_J = 0.08$ with $p_1 = 0.05$. Prior distributions: logit: $\beta_0 \sim N(-1.073, 1.128)$, $\beta_1 \sim N(0.687, 0.500)$; log-log: $\beta_0 \sim N(-0.196, 1.217)$, $\beta_1 \sim N(0.328, 0.569)$; clog-log: $\beta_0 \sim N(-1.255, 0.932)$, $\beta_1 \sim N(0.092, 0.642)$.

Dose levels		1	2	3	4	5	6
Doses		10	20	30	45	60	80
scenario 1	PAVA	0.9	9.2	28.1	**33.8**	24.6	3.4
	logit	0.0	7.5	31.2	**41.2**	18.8	1.3
	log-log	0.0	9.5	32.9	**40.4**	16.2	1.0
	clog-log	0.0	7.3	29.7	**41.3**	19.7	2.0
scenario 2	PAVA	0.0	0.7	7.4	14.6	26.6	**50.7**
	logit	0.0	0.0	7.2	18.0	31.2	**43.6**
	log-log	0.0	0.0	7.6	19.6	31.0	**41.8**
	clog-log	0.0	0.0	6.9	16.9	29.4	**46.8**
scenario 3	PAVA	0.0	0.2	6.9	27.4	**56.7**	8.8
	logit	0.0	0.0	4.8	37.4	**52.3**	5.5
	log-log	0.0	0.0	5.7	36.5	**52.9**	4.9
	clog-log	0.0	0.0	4.2	35.7	**52.8**	7.3
scenario 4	PAVA	19.6	**46.8**	27.2	4.5	0.8	0.0
	logit	7.9	**58.1**	28.3	4.5	0.1	0.0
	log-log	15.7	**56.8**	23.8	2.5	0.1	0.0
	clog-log	7.2	**57.0**	29.8	4.8	0.1	0.0
scenario 5	PAVA	3.1	29.2	**51.0**	14.1	2.4	0.1
	logit	0.2	26.1	**58.8**	14.0	0.8	0.0
	log-log	1.2	32.9	**53.3**	11.7	0.8	0.0
	clog-log	0.2	24.3	**59.9**	14.6	0.9	0.0
scenario 6	PAVA	0.2	2.6	26.3	**49.4**	19.6	1.9
	logit	0.0	0.9	27.3	**57.2**	13.8	0.8
	log-log	0.0	1.4	30.9	**54.6**	12.5	0.6
	clog-log	0.0	0.9	25.2	**56.9**	16.0	1.0
scenario 7	PAVA	20.0	**61.9**	16.9	1.1	0.0	0.0
	logit	1.7	**75.6**	22.3	0.3	0.0	0.0
	log-log	6.7	**76.4**	16.5	0.3	0.0	0.0
	clog-log	1.6	**74.4**	23.4	0.5	0.0	0.0
scenario 8	PAVA	3.1	29.5	**52.7**	13.3	1.3	0.0
	logit	0.2	26.2	**61.4**	11.9	0.2	0.0
	log-log	1.2	33.4	**55.3**	9.8	0.2	0.0
	clog-log	0.2	24.4	**62.2**	12.8	0.3	0.0

Table A.6: MTD selection results for $p_J = 0.11$ with $p_1 = 0.05$. Prior distributions: logit: $\beta_0 \sim N(-0.939, 1.407)$, $\beta_1 \sim N(0.565, 0.805)$; log-log: $\beta_0 \sim N(-0.095, 0.906)$, $\beta_1 \sim N(0.085, 0.520)$; clog-log: $\beta_0 \sim N(-1.391, 0.939)$, $\beta_1 \sim N(0.310, 0.635)$.

Dose levels		1	2	3	4	5	6
Doses		10	20	30	45	60	80
scenario 1	PAVA	0.9	9.2	28.1	**33.8**	24.6	3.4
	logit	0.0	8.2	29.6	**39.5**	19.6	3.1
	log-log	0.0	8.8	32.8	**40.8**	16.6	1.0
	clog-log	0.0	7.2	29.2	**40.0**	20.5	3.1
scenario 2	PAVA	0.0	0.7	7.4	14.6	26.6	**50.7**
	logit	0.0	0.0	7.7	15.6	27.1	**49.6**
	log-log	0.0	0.0	7.4	19.2	31.0	**42.4**
	clog-log	0.0	0.0	7.2	15.6	27.5	**49.7**
scenario 3	PAVA	0.0	0.2	6.9	27.4	**56.7**	8.8
	logit	0.0	0.0	6.0	31.9	**53.1**	9.0
	log-log	0.0	0.0	5.0	37.3	**52.4**	5.3
	clog-log	0.0	0.0	5.0	32.3	**53.4**	9.3
scenario 4	PAVA	19.6	**46.8**	27.2	4.5	0.8	0.0
	logit	11.3	**56.2**	26.8	4.5	0.1	0.0
	log-log	13.5	**57.9**	25.1	2.3	0.1	0.0
	clog-log	7.1	**55.3**	31.4	5.0	0.1	0.0
scenario 5	PAVA	3.1	29.2	**51.0**	14.1	2.4	0.1
	logit	0.4	28.1	**55.9**	14.5	0.9	0.1
	log-log	0.5	32.2	**55.0**	11.4	0.8	0.0
	clog-log	0.1	23.5	**59.4**	15.8	1.0	0.1
scenario 6	PAVA	0.2	2.6	26.3	**49.4**	19.6	1.9
	logit	0.0	1.4	26.2	**54.8**	16.0	1.6
	log-log	0.0	1.0	30.0	**55.6**	13.0	0.4
	clog-log	0.0	0.9	24.8	**55.8**	16.9	1.6
scenario 7	PAVA	20.0	**61.9**	16.9	1.1	0.0	0.0
	logit	3.3	**76.2**	19.9	0.5	0.0	0.0
	log-log	4.9	**77.4**	17.3	0.3	0.0	0.0
	clog-log	1.6	**72.7**	24.9	0.7	0.0	0.0
scenario 8	PAVA	3.1	29.5	**52.7**	13.3	1.3	0.0
	logit	0.4	28.3	**58.2**	12.6	0.4	0.0
	log-log	0.5	32.6	**57.0**	9.7	0.1	0.0
	clog-log	0.1	23.5	**61.5**	14.4	0.4	0.0

Table A.7: MTD selection results for $p_J = 0.3$ with $p_1 = 0.05$. Prior distributions: logit: $\beta_0 \sim N(-1.392, 1.358)$, $\beta_1 \sim N(0.322, 0.500)$; log-log: $\beta_0 \sim N(-0.229, 1.128)$, $\beta_1 \sim N(0.249, 0.500)$; clog-log: $\beta_0 \sim N(-1.655, 0.993)$, $\beta_1 \sim N(-0.140, 0.655)$.

Dose levels		1	2	3	4	5	6
Doses		10	20	30	45	60	80
scenario 1	PAVA	0.9	9.2	28.1	**33.8**	24.6	3.4
	logit	0.0	6.8	26.5	**41.8**	21.3	3.6
	log-log	0.0	9.2	32.7	**40.9**	16.2	1.0
	clog-log	0.0	6.2	25.2	**39.9**	23.3	5.4
scenario 2	PAVA	0.0	0.7	7.4	14.6	26.6	**50.7**
	logit	0.0	0.0	5.8	16.0	26.3	**51.9**
	log-log	0.0	0.0	7.5	19.7	31.0	**41.8**
	clog-log	0.0	0.0	5.9	15.0	24.0	**55.1**
scenario 3	PAVA	0.0	0.2	6.9	27.4	**56.7**	8.8
	logit	0.0	0.0	3.1	30.1	**55.5**	11.3
	log-log	0.0	0.0	5.2	36.8	**52.9**	5.1
	clog-log	0.0	0.0	3.1	27.8	**54.1**	15.0
scenario 4	PAVA	19.6	**46.8**	27.2	4.5	0.8	0.0
	logit	8.6	**56.0**	29.1	5.1	0.1	0.0
	log-log	15.4	**57.0**	23.9	2.5	0.1	0.0
	clog-log	6.3	**55.0**	31.5	5.7	0.4	0.0
scenario 5	PAVA	3.1	29.2	**51.0**	14.1	2.4	0.1
	logit	0.2	23.5	**58.3**	16.7	1.1	0.1
	log-log	1.0	32.5	**53.7**	11.9	0.8	0.0
	clog-log	0.1	21.0	**57.0**	19.6	2.1	0.1
scenario 6	PAVA	0.2	2.6	26.3	**49.4**	19.6	1.9
	logit	0.0	0.9	20.8	**57.8**	18.9	1.6
	log-log	0.0	1.0	30.2	**55.4**	13.0	0.4
	clog-log	0.0	0.9	19.5	**55.4**	21.7	2.5
scenario 7	PAVA	20.0	**61.9**	16.9	1.1	0.0	0.0
	logit	2.0	**74.0**	23.1	0.8	0.0	0.0
	log-log	6.0	**76.8**	16.8	0.3	0.0	0.0
	clog-log	1.5	**71.9**	25.3	1.2	0.0	0.0
scenario 8	PAVA	3.1	29.5	**52.7**	13.3	1.3	0.0
	logit	0.2	23.7	**60.5**	14.8	0.7	0.0
	log-log	1.0	33.0	**55.7**	10.0	0.2	0.0
	clog-log	0.1	21.1	**59.5**	18.2	1.0	0.0

A.2 Large variance prior for coefficient parameters

We examined the impact of using large variance normal priors for the coefficient parameters β_0 and β_1 in the dose-response model (1). This analysis contrasts with the main simulation results (Table 4 in the main manuscript), which employed carefully specified priors derived through our proposed approach in Section 3.2. Specifically, we replaced our default prior specifications, derived in Section 4.2, with normal distributions having large variances (variance = 10), while maintaining the same mean values. This represents approximately 10-fold increases in variance compared to the carefully calibrated specifications used in the main analysis. For example, for the logit link function, the large variance priors were: $\beta_0 \sim N(-1.592, 10)$, $\beta_1 \sim N(0.412, 10)$ compared to the default specification $\beta_0 \sim N(-1.592, 1.371)$, $\beta_1 \sim N(0.412, 0.784)$. This large variance specification leads to U-shaped prior distributions for DLT probabilities at intermediate dose levels, where probability mass concentrates near the extremes (0 and 1) rather than moderate values typically expected in clinical practice.

Table A.8 presents the MTD selection results under this prior specification across all eight scenarios. Comparing these results with Table 4 in the main manuscript reveals several important patterns. The large variance priors demonstrate markedly different performance depending on the location of the true MTD. In scenarios where the true MTD lies at boundary dose levels (e.g., Scenario 2 with MTD at the highest dose level 6), the large variance priors sometimes yield favorable results. For example, in Scenario 2, the logit model with large variance priors achieves 56.2% correct MTD selection compared to 51.6% with our default specification, representing a 4.6 percentage point improvement. This occurs because the U-shaped prior distribution, which concentrates probability mass at extreme values (near 0 and 1), aligns well with scenarios where the MTD is expected to be at the boundary.

However, in all scenarios except Scenario 2, where the MTD is at the highest dose, the large variance priors consistently deteriorate performance. For example, in Scenario 1 (MTD at dose level 4), the proportion of correct MTD selections for the logit model drops from 39.5% with default priors to 34.3% with large variance priors, representing a 5.2 percentage point decrease. More substantial reductions exceed 10 percentage points in Scenarios 5 and 8, where the proportions drop from 57.9% to 47.5% and from 60.1% to 49.4%, respectively. Overall, across all seven scenarios with intermediate MTDs, the large variance priors result in an average decrease of 7.5 percentage points in MTD selection accuracy for the logit model, with similar deterioration patterns consistently observed across all three link functions (logit, log-log, and complementary log-log). This demonstrates that the adverse effects of inappropriate prior specification are not limited to a particular modeling choice but represent a fundamental issue with U-shaped prior distributions for intermediate dose levels. The deterioration occurs because the U-shaped prior distribution conflicts with the clinical expectation of moderate toxicity rates at intermediate dose levels, where the true MTD typically resides in Phase I oncology trials.

Table A.8: The proportion of MTD selections across 1,000 simulations in each scenario for the PAVA-based method and the proposed methods using the logit, log-log, and complementary log-log models. The optimal MTD is highlighted in bold.

Dose levels		1	2	3	4	5	6
Doses		10	20	30	45	60	80
scenario 1	PAVA	0.9	9.2	28.1	**33.8**	24.6	3.4
	logit	0.8	7.7	24.7	**34.3**	23.7	8.8
	log-log	0.8	8.5	24.6	**35.0**	22.9	8.2
	clog-log	0.8	7.5	24.8	**33.0**	25.3	8.6
scenario 2	PAVA	0.0	0.7	7.4	14.6	26.6	**50.7**
	logit	0.0	0.4	6.9	14.0	22.5	**56.2**
	log-log	0.0	0.5	6.9	14.1	23.0	**55.5**
	clog-log	0.0	0.4	6.6	13.9	22.7	**56.4**
scenario 3	PAVA	0.0	0.2	6.9	27.4	**56.7**	8.8
	logit	0.0	0.1	5.5	25.7	**52.8**	15.9
	log-log	0.0	0.1	5.8	27.1	**52.4**	14.6
	clog-log	0.0	0.1	5.1	24.3	**54.0**	16.5
scenario 4	PAVA	19.6	**46.8**	27.2	4.5	0.8	0.0
	logit	18.3	**47.6**	25.2	6.9	0.9	0.0
	log-log	21.8	**46.5**	24.0	5.7	0.9	0.0
	clog-log	18.4	**45.2**	27.1	7.1	1.1	0.0
scenario 5	PAVA	3.1	29.2	**51.0**	14.1	2.4	0.1
	logit	3.3	26.1	**47.5**	19.3	3.5	0.2
	log-log	3.8	28.0	**47.1**	17.3	3.5	0.2
	clog-log	3.3	25.3	**47.1**	20.6	3.4	0.2
scenario 6	PAVA	0.2	2.6	26.3	**49.4**	19.6	1.9
	logit	0.2	2.0	21.2	**48.4**	23.2	5.0
	log-log	0.2	2.4	22.2	**49.1**	21.4	4.7
	clog-log	0.2	2.0	20.1	**47.6**	25.0	5.1
scenario 7	PAVA	20.0	**61.9**	16.9	1.1	0.0	0.0
	logit	16.7	**63.9**	17.9	1.3	0.1	0.0
	log-log	18.5	**63.1**	17.1	1.1	0.1	0.0
	clog-log	16.0	**62.6**	19.9	1.3	0.1	0.0
scenario 8	PAVA	3.1	29.5	**52.7**	13.3	1.3	0.0
	logit	3.3	26.4	**49.4**	18.7	2.1	0.0
	log-log	3.9	28.4	**48.5**	17.3	1.8	0.0
	clog-log	3.3	25.6	**49.1**	19.7	2.2	0.0

References

Neuenschwander, B., Branson, M., and Gsponer, T. (2008). Critical aspects of the bayesian approach to phase I cancer trials. *Statistics in medicine*, 27(13):2420–2439.
